# Update: Ebola Virus Disease Outbreak — West Africa, October 2014

**Published:** 2014-10-31

**Authors:** Incident Management System Ebola Epidemiology Team, CDC; Guinea Interministerial Committee for Response Against the Ebola Virus; CDC Guinea Response Team; Liberia Ministry of Health and Social Welfare; CDC Liberia Response Team; Sierra Leone Ministry of Health and Sanitation; CDC Sierra Leone Response Team; Viral Special Pathogens Branch, National Center for Emerging and Zoonotic Infectious Diseases, CDC

CDC is assisting ministries of health and working with other organizations to control and end the ongoing outbreak of Ebola virus disease (Ebola) in West Africa (1). The updated data in this report were compiled from situation reports from the Guinea Interministerial Committee for Response Against the Ebola Virus and the World Health Organization, the Liberia Ministry of Health and Social Welfare, and the Sierra Leone Ministry of Health and Sanitation. Total case counts include all suspected, probable, and confirmed cases as defined by each country. These data reflect reported cases, which make up an unknown proportion of all actual cases and reporting delays that vary from country to country.

According to the latest World Health Organization update as of October 22, 2014 (2), a total of 9,911 Ebola cases have been reported as of October 19 from three highly affected West African countries (Guinea, Liberia, and Sierra Leone) (Figure 1). The highest reported case counts were from Liberia (4,665 cases), followed by Sierra Leone (3,706) and Guinea (1,540).

The geographic distribution of the number of Ebola cases reported during September 28–October 18 changed from the distribution of cases reported during August 31–September 23 (3), when counts were highest in the areas where Liberia, Sierra Leone, and Guinea meet. Counts of Ebola cases reported during September 28–October 18 were highest in the area around Monrovia and in the district of Bong, Liberia; the Freetown area and the northwest districts of Sierra Leone; and the district of Macenta, Guinea (Figure 2).

The map of the cumulative incidence of Ebola, as of October 18, indicates that the highest incidence rate (>100 cases per 100,000 population) was reported by two districts in Guinea (Guéckédou and Macenta), five districts in Liberia (Bomi, Bong, Lofa, Margibi, and Montserrado), and four districts in Sierra Leone (Bombali, Kailahun, Kenema, and Port Loko) (Figure 3).

The latest updates on the 2014 Ebola outbreak in West Africa, including case counts, are available at http://www.cdc.gov/vhf/ebola/outbreaks/guinea/index.html. The most up-to-date clinical guidelines on the 2014 Ebola outbreak in West Africa are available at http://www.cdc.gov/vhf/ebola/hcp/index.html.

## Figures and Tables

**FIGURE 1:**
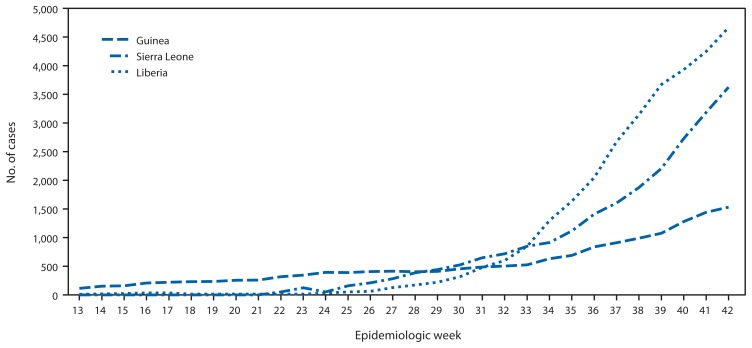
Cumulative number of Ebola virus disease cases reported, by epidemiologic week — three countries, West Africa, March 29–October 18, 2014

**FIGURE 2:**
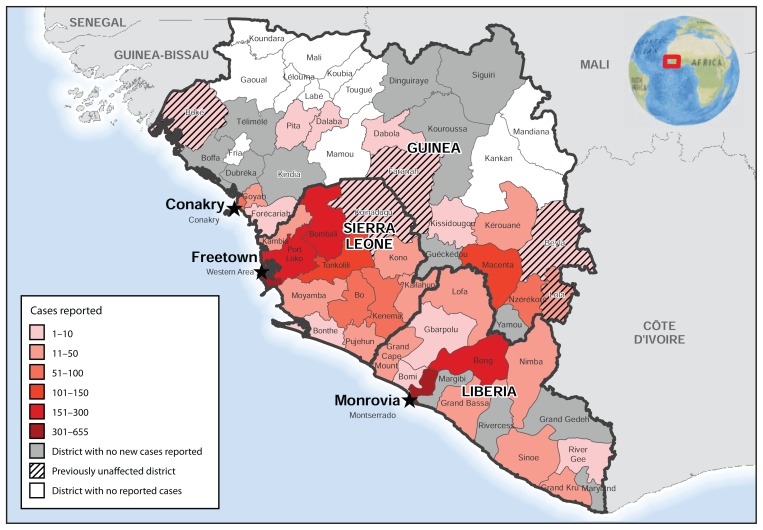
Number of new cases of Ebola virus disease reported — West Africa, September 28–October 18

**FIGURE 3:**
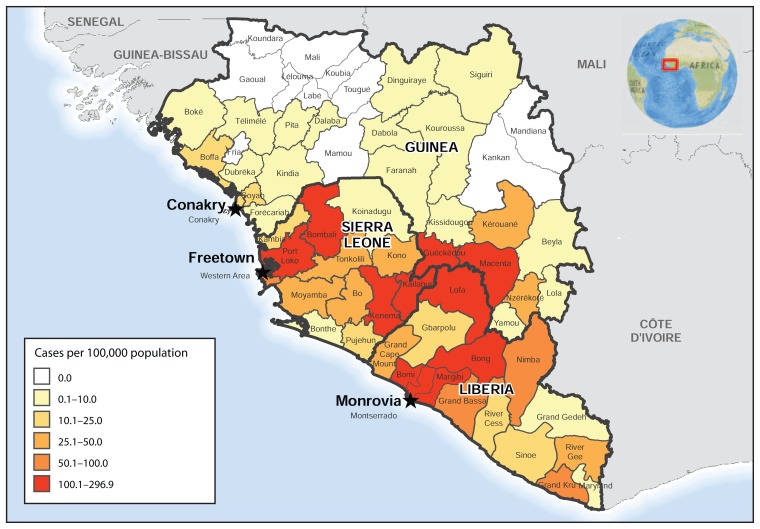
Ebola virus disease cumulative incidence — West Africa, October 18, 2014* * Cumulative number of reported Ebola virus disease cases per 100,000 persons since December 22, 2013.
